# From General Principles to Procedural Values: Responsible Digital Health Meets Public Health Ethics

**DOI:** 10.3389/fdgth.2021.690417

**Published:** 2021-07-02

**Authors:** Rune Nyrup

**Affiliations:** Leverhulme Centre for the Future of Intelligence, University of Cambridge, Cambridge, United Kingdom

**Keywords:** digital ethics, principlism, public health ethics, procedural values, accountability for reasonableness, A4R

## Abstract

Most existing work in digital ethics is modeled on the “principlist” approach to medical ethics, seeking to articulate a small set of general principles to guide ethical decision-making. Critics have highlighted several limitations of such principles, including (1) that they mask ethical disagreements between and within stakeholder communities, and (2) that they provide little guidance for how to resolve trade-offs between different values. This paper argues that efforts to develop responsible digital health practices could benefit from paying closer attention to a different branch of medical ethics, namely public health ethics. In particular, I argue that the influential “accountability for reasonableness” (A4R) approach to public health ethics can help overcome some of the limitations of existing digital ethics principles. A4R seeks to resolve trade-offs through decision-procedures designed according to certain shared procedural values. This allows stakeholders to recognize decisions reached through these procedures as legitimate, despite their underlying disagreements. I discuss the prospects for adapting A4R to the context of responsible digital health and suggest questions for further research.

## Introduction

Recent years have seen a proliferation of digital ethics guidelines. There now exist more than 160 such guidelines, the vast majority published within the last 5 years by a wide range of institutions, including governments, legislative bodies, technology companies, and academic and professional organizations (1). These guidelines are intended for a number of purposes, including as a guide for designers of new digital technologies, to identify and address issues arising from the deployment of such technologies, and as a basis for developing standards and regulation (2).

Many seeking to bring analytical clarity to this panoply have looked to medical ethics for inspiration (3, 4). This is unsurprising: medical ethics is perhaps the most well-established field of practical ethics, both within academic research and as a framework for practitioners. For digital health technologies there is of course the additional reason that they are designed to become part of medical practice. Responsible digital health should involve being held to the same ethical standards as any other form of medical practice (5).

Most of this work has been modeled on an approach to medical ethics known as “principlism.” Principlism seeks to articulate a small set of general principles to guide ethical decision-making. Most influentially, Tom Beauchamp & James Childress' four *Principles of Biomedical Ethics* (6)—Beneficence, Non-Maleficence, Autonomy and Justice—are widely used and taught within clinical practice and research ethics. Many reviews of digital ethics guidelines similarly seek to subsume their recommendations under a small set of general principles, and some explicitly use Beauchamp & Childress' four principles (sometimes with a new fifth principle of Explicability) (3, 7–10). The convergence on these principles is often touted as evidence of an emerging consensus which can serve as a basis for implementing ethics into the design, regulation, and application of digital technologies. Yet how this is to be done largely remains an open question (11). Consequently, digital ethicists have increasingly turned their attention to how such principles can best be translated into practice, whether through new design practices (5, 12, 13) or new forms of legislation and regulation (14, 15).

However, critics have highlighted several limitations which vitiate the practical applicability of this approach to digital ethics (2, 9, 16–18). In this paper, I focus on two in particular. First, principles formulated in general, abstract terms mask underlying disagreements between and within stakeholder communities. Second, they provide little guidance for how to resolve tensions and trade-offs that can arise between different (interpretations of) principles. To overcome these limitations, I argue, efforts to develop more responsible digital health practices should pay closer attention to a different branch of medical ethics: public health ethics.

I start by making a general case for this claim. I then discuss the problems of disagreement and trade-offs within digital ethics, before introducing an influential account from public health ethics of how to reach ethically legitimate compromises on value-laden trade-offs. This approach, known as accountability for reasonableness (A4R) is based on the idea that legitimate compromises can be reached through decision-procedures designed according to certain procedural values (19). Finally, I discuss the prospects for adapting this approach to digital health and propose some questions for future research.

## Why Public Health Ethics?

Public health differs from clinical practice in two key respects (20): in who is affected, and in who decides and implements interventions. Public health interventions affect broader populations, rather than specific, identifiable patients, and they are largely decided and implemented by institutional actors (e.g., governments, insurance companies, NGOs), rather than individual clinicians/researchers.

There are two general reasons why closer attention to public health ethics is likely to benefit efforts to develop responsible digital health.

First, digital health technologies are often similar to public health interventions. Some are explicitly designed for public health purposes, such as monitoring infectious disease outbreaks (21, 22) or discovering risk factors for childhood obesity (23). But many digital technologies deployed in clinical settings also resemble public health interventions. Take machine learning tools for diagnostic decision-support (24, 25). These are usually designed for screening purposes, to monitor data from a given patient population and flag risk factors to human clinicians, and decisions to deploy them are made at the institutional level (e.g., hospitals or health service trusts). Even in patient-facing applications, e.g., conversational agents to assist with lifestyle decisions (26), many of the pertinent ethical decisions have to be made at the population/institutional level—by designers and regulators—rather than in the individual clinical encounter.

The second reason follows from the first. Due to its focus on population/institution-level interventions, public health ethics mainly addresses questions of political morality rather than the ethics of the individual patient-clinician relationship (20). It therefore provides a promising resource for addressing important political issues that arise from digital health.

Recent digital ethics has mostly focused on technological deficiencies and solutions, such as algorithmic bias and transparency. As several commentators have highlighted, this risks occluding broader social and political issues relating, e.g., to democratic oversight, power, and oppression (27–33). For example, it was recently shown that an algorithm that uses healthcare costs as a proxy for healthcare needs systematically underestimated the needs of Black patients, because less resources are already spent on their care (34). Ruha Benjamin (35) argues that labeling this “algorithmic bias,” makes it seem a purely technical issue and sanitizes the social context that produced the problem in the first place, namely persistent structural and interpersonal racism in healthcare. More generally, as Leila Marie Hampton (30) argues, using generic concepts such as “fairness” or “transparency” to analyze technologies, without considering broader socio-political issues, risks legitimizing, and entrenching fundamentally unjust institutions.

While the Four Principles do include a principle of Justice, political issues covered under this heading mainly concern the question of what health-related goods society should provide and how to allocate resources within healthcare systems (5, chapter 6). By contrast, public health interventions raise a much wider set of political issues (20), similar to those commentators have started to discuss for digital health. For instance, is it permissible for interventions to impose risks or burdens on some individuals, even if they are not the main beneficiaries (e.g., mandatory vaccination programs)? Is it justifiable for interventions to exploit or reinforce structural patterns of disadvantage (e.g., using the communicative power of the state to stigmatize smoking)? More generally, when can institutional actors legitimately impose interventions despite widespread disagreement about relevant ethical values?

To be clear, my aim is not to reject the Four Principles framework or other principlist approaches to digital ethics. Such principles still serve a useful purpose in articulating the values at stake in digital ethics (cf. Section **What rationales should be considered relevant?)**. Similarly, public health ethics will not, in itself, answer all of the socio-political issues that Benjamin, Hampton and others raise. Clearly, many of these require political action and structural change, not (just) better theory. Even in terms of theory, other literatures will be relevant too, especially emancipatory philosophies such as the Black Feminist tradition Hampton highlights. Nonetheless, public health ethics is a well-developed literature addressing practical political issues in healthcare, often closely informed by the empirical realities of healthcare policy and decision-making. It can thus help broaden the range of questions digital health ethics addresses.

## Disagreement, Trade-Offs, and the Limits of Principles

The rest of this paper will focus on how insights from public health ethics can help overcome the two limitations of purely principlist approaches to digital ethics I highlighted in the introduction, i.e., that they mask disagreements between and within different stakeholder communities and provide little guidance for how to resolve trade-offs.

Consider for example debates about contact tracing apps for the management of Covid-19. Some governments wanted to base these on a centralized data collection approach, arguing that such datasets could also be used to produce new knowledge to help combat the pandemic. This was resisted by legal and information security experts concerned about potential privacy breaches (36–38). Appealing to general principles is unlikely to resolve this debate. While most people would presumably agree, say, that digital health technologies should be used to “do good” (Beneficence), there are legitimate ethical and political disagreements about the extent to which privacy is constitutive of or conducive to a good life. While we should arguably accept some trade-offs between protecting individual privacy and promoting social goods, there is little consensus on what exactly those trade-offs should be (38).

The prevalent approach to managing value trade-offs within clinical ethics is through informed consent (5, chapter 3): by informing patients about the trade-off involved in some treatment and letting them decide whether this is acceptable in light of their particular circumstances and values, clinicians can legitimize the decision to administer or withhold the treatment. It might be tempting to apply the same approach to digital health. However, informed consent is only plausible when the trade-offs occur within a single patient's value-set. One of the ways digital health resembles public health is that the trade-offs often cut across populations. Rather than each patient deciding for themselves how to balance trade-offs, which values get priority depends on population-level aggregate decisions. Contact tracing apps, and centralized data collection more generally, can only produce the relevant social goods if there is sufficient uptake (39). Conversely, if enough people consent to share their personal data, this can often be used to train machine learning algorithms capable of inferring highly personal information even about those who withhold consent (40).

In such cases, making interventions conditional on obtaining everyone's consent is neither practically feasible nor ethically plausible. A single intransigent individual should not be allowed to deprive everyone else of significant social goods. However, pure majority rule is not plausible either. Certain groups and communities may have good reasons, e.g., to value privacy because of their historical experiences of surveillance and discrimination (37). For instance, during the 1980's AIDS crisis, gay community-based activists initially resisted name-based reporting of infections, arguing that homophobia and AIDS-hysteria made privacy breaches and discrimination against people identified as HIV-positive more likely than for other diseases (41). Even if such reasons should not necessarily be decisive, collective decision-making should at least be responsive to them, and not just defer to majority preferences.

## Legitimacy Through Procedural Values

How to resolve disagreement and trade-offs is a characteristic conundrum in public health ethics. For example, in debates about priority setting and rationing of healthcare resources, ethicists have found it difficult to formulate ethical principles that are plausible enough to command broad consensus while being sufficiently fine-grained to guide decision-making in practice (42, 43). While many agree that those with greater needs should be given some priority, even at the expense of aggregate health outcomes, there is little consensus on how to weigh these two concerns against each other.

One influential model for resolving disagreements about priority setting in public health is called Accountability for Reasonableness (A4R) (19, 44, 45). Proposed by Norman Daniels and James Sabin, the key idea in A4R is to implement decision-procedures for reaching compromises which fair-minded people can accept as legitimate, despite their underlying ethical disagreements. This relies on a distinction between ethical *rightness* and ethical *legitimacy*. To regard a decision as right is to regard it as the morally correct thing to do in a given situation. To regard it as legitimate is to regard it as appropriately made, i.e., by a decision-maker or procedure whose moral authority to make such decisions should be accepted. The two can come apart: we can accept a verdict of “not guilty” in a fair trial as legitimate, even if we believe the defendant should have been convicted. Conversely, an unelected dictator may sometimes do the right thing, e.g., donate food to relieve a famine. Nonetheless, rightness and legitimacy are also entangled: if a procedure consistently generates abhorrent outcomes, we have reason to question its legitimacy; and if we can see that a decision-maker has carefully considered the relevant concerns, there is prima facie reason to accept their decision as right.

Daniels and Sabin propose four conditions for legitimate decision-procedures (44, 45):

1. *Publicity:* The rationale for a given decision must be publicly accessible.
2. *Relevance:* Decisions must be based on rationales which fair-minded individuals, who want to find mutually justifiable terms of cooperation, would accept as relevant to the decision.
3. *Revision and Appeals:* There must be mechanisms in place for challenging and revising decisions in light of new evidence or arguments.
4. *Enforcement:* There must be voluntary or public regulation in place to ensure that conditions 1–3 are met.

These conditions can be interpreted as embodying certain *procedural values*, specifying features that fair and appropriate decision procedures should have. It is a shared commitment to procedural values that generates legitimacy. Stakeholders who agree on these values have good reasons to regard procedures designed according to them as legitimate.

As the name suggests, the core procedural values in A4R are Accountability and Reasonableness. By articulating standards and mechanisms that stakeholders can use to hold decision-makers *accountable*—through enforceable rights to access rationales and challenge decisions—A4R aims to produce decisions that are *reasonable*, and can be recognized as such. Reasonableness here means something weaker than rightness: a decision is reasonable to the extent that it is responsive to all relevant concerns. Thus, if you recognize a decision as reasonable you may disagree about the specific way decision-makers weighed the reasons cited in their rationale, but you agree that it involved the right kinds of considerations.

The A4R conditions are supposed to guide the design of decision-making bodies charged with deciding how to balance any trade-offs that arise within a given healthcare institution (e.g., a hospital, public health agency or insurance company). Decision-makers should strive to identify compromises which all fair-minded stakeholders could find acceptable, though, some form of voting may be used if disagreement persists at the end of deliberation. Importantly, decision-makers do not need to articulate any general hierarchy of values or “meta-principles” for resolving trade-offs. Indeed, one of the motivations behind A4R is that we are unlikely to agree on any sufficiently action-guiding meta-principles. Rather, it aims to resolve trade-offs on a case-by-case basis as they arise in practice, based on rationales stakeholders will find contextually reasonable, despite persistent disagreement about general principles.

A4R is not without its detractors (little in philosophy is), nor is it the only account in public health ethics of how to resolve trade-offs (20). Nonetheless, it is a highly influential framework which has been used to inform public health practice (46, 47) and whose acceptability to decision-makers has been studied empirically across the world (48–50). Furthermore, public health ethicists have proposed a number of revisions and extensions of the A4R framework, reflecting lessons from these practical applications (51–54). As such, the A4R literature is likely to contain valuable lessons for responsible digital health^1^.

## Adapting A4R to Digital Health

In the Introduction I highlighted two routes that ethicists have proposed for translating existing principles into practice: legislation/regulation and design practices. A4R can help overcome some of the limitations of the principlist approach within each of these.

Regarding the first, the challenge is to translate abstract general principles into more concrete legislation and regulation while still preserving their broad appeal. However, attempts to make principles more concrete and action-guiding, including any meta-principles for resolving trade-offs, will likely also make them more controversial. The A4R framework provides an alternative solution: rather than having to settle on a specific action-guiding translation of principles, legislators can instead specify how organizations that deploy or design digital health technologies should structure the decision-making processes through which they resolve any trade-offs they encounter.

As mentioned, deliberative bodies based on the A4R conditions have already been implemented in some healthcare institutions to address issues of priority setting and rationing. The remit of these could be expanded to also address the broader range of trade-offs that arise from the deployment of digital health technologies. Legislators could also require decision-making bodies modeled on the existing ones to be created elsewhere, including within private technology companies or as part of regulators charged with overseeing them.

Whether legally required or voluntarily adopted, this type of deliberative body could also provide a way to deal with trade-offs in the design of digital health technologies. A common criticism of Value-Sensitive Design (VSD) is that it lacks a method for resolving trade-offs, except if designers commit to an explicit – and therefore likely controversial – ethical theory (56, 57). This challenge will also affect proposals to implement digital ethics principles through (a modified version of) VSD (12). A4R suggests a way to overcome it: by structuring their decision-making processes according to the right kinds of procedural values, designers will be able to reach decisions that stakeholders can recognize as legitimate and therefore acceptable. To be clear, A4R is a *normative* theory of legitimacy. It does not commit the naturalistic fallacy by assuming that whatever stakeholders find acceptable is therefore right. If a decision counts as legitimate, according to A4R, stakeholders *ought* to find it acceptable.

## Future Research Questions

There are of course many details to be worked out regarding the proposals sketched here. How to best implement and operationalize them in practice remains an important question for future research. Part of this will practical, but A4R also provides a philosophically grounded theory to underpin this research and ensure that proposed implementations remain normatively plausible.

However, we should not expect that A4R can simply be transposed from its original application (priority setting and rationing) to digital health without modification. Adapting A4R to digital health will likely require modifications or extensions to the framework itself. At least two kinds of further research questions will be relevant to explore.

### Are Other Procedural Values Needed?

One of the ways public health ethicists have extended the original A4R framework is by adding further procedural values, often motivated by their practical experience of applying A4R to priority setting decisions. For instance, some have proposed new conditions of Inclusiveness and Empowerment. In brief, these require explicit input from all affected stakeholders and that active steps are taken to counteract knowledge-gaps and institutional power differences between decision-makers (33, 53, 58). Importantly, these conditions are still motivated by the core value of Reasonableness, namely to ensure that decision-makers are responsive to as many relevant concerns as possible, including those that are held by minoritized or less empowered parts of the population.

Applying A4R to digital health may similarly reveal new procedural values. For instance, if Benjamin and Hampton are correct that ethical discussions of digital technologies risk sanitizing and entrenching unjust social structures, it may be necessary to actively encourage decision-makers to raise critical questions about how new technologies will interact with these structures. Similarly, it may be necessary to encourage scrutiny of the aims and presuppositions of the technology itself, asking for example whether it targets the right problem or whether the proposed solution is at all appropriate. We might summarize these as a condition of Socio-Technological Criticism.

### What Rationales Should Be Considered Relevant?

The Relevance condition is a formal constraint on the type of rationales that should be given weight within decision-making. However, implementing A4R in practice requires us to specify in more substantive terms what types of concerns should be admissible. This will likely depend on the context of application. As A4R was originally developed for debates about rationing, most discussions focus on rationales framed in terms of Fairness or related distributive values (e.g., Solidarity (52)). Presumably, a broader range of values will be relevant to debates about digital health technologies (e.g., Privacy). Exploring in more detail what those values should be is a substantive research task. To ensure that decision-makers are responsive to all relevant reasons, this research should aim to identify a broad range of plausible concerns and help elucidate and articulate these, so that stakeholders can present them in their most compelling form. Existing VSD methodologies for empirical and conceptual investigations of stakeholder values provide a plausible approach to this task.

Existing principlist approaches to digital ethics provide a useful starting point. However, the values discussed in the existing literature should not be assumed exhaustive or representative. The apparent convergence found here may simply be a product of people from roughly similar backgrounds consuming the same literature (2, 17). It is noticeable, for instance, that many commonly cited principles (e.g., transparency, fairness, responsibility) also feature prominently within liberal political philosophy. Values more characteristic of other political traditions, such as solidarity, belonging, authenticity, harmony, non-exploitation, non-domination or emancipation are rarely discussed or even mentioned (9, 29, 30). Public health ethics may also here provide a useful resource. Public health ethicists have developed alternative sets of principles to the four classical principles of biomedical ethics (59), and explored the implications of different political traditions (60).

## Conclusion

Paying closer attention to public health ethics is likely to benefit efforts to develop responsible digital health. In this paper, I have made a general case for this claim and highlighted A4R as a specific model from public health ethics that can be adapted to digital health. While not intended to wholly replace principlism, A4R can complement and help overcome some of the limitations faced by principlist approaches. Further, research on the questions outlined above could generate valuable insights for the ethical deployment, design and regulation of digital technologies, especially within healthcare.

## Data Availability Statement

The original contributions presented in the study are included in the article/supplementary material, further inquiries can be directed to the corresponding author/s.

## Author Contributions

The author confirms being the sole contributor of this work and has approved it for publication.

## Conflict of Interest

The author declares that the research was conducted in the absence of any commercial or financial relationships that could be construed as a potential conflict of interest.

## Footnotes

^1^To my knowledge, only two other recent papers have discussed the application of A4R to digital (health) ethics (33, 55), though not along the same lines as me.
